# Supplementary material for: Structural host-virus interactome profiling of intact infected cells
Source: Nat Commun. 2025 Jul 21;16:6713. doi: 10.1038/s41467-025-61618-z (PMC12280212; doi:10.1038/s41467-025-61618-z)
Supplement: Supplementary file 1 — Supplementary Information [file 41467_2025_61618_MOESM1_ESM.pdf]

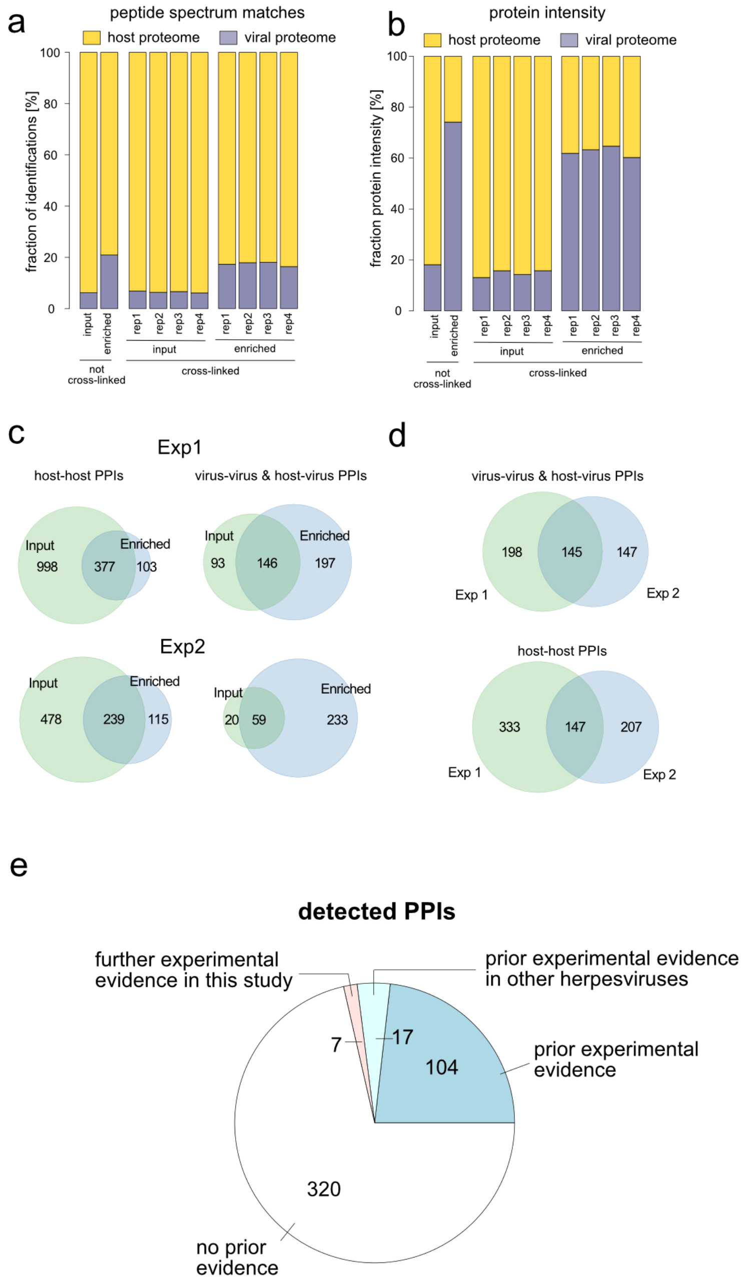

Supplementary Figure 1. SHVIP quality control. (a,b) *The number of peptide spectrum matches (a) and overall protein intensity (b) from an analysis of the linear peptides by LC-MS/MS with indicated treatments.* ***(c)*** *Overlap of PPI identifications in enriched and input samples after cross-link analysis in both experiments. Experiment 1 was performed in MS2-MS3 and experiment 2 in an MS2 only acquisition scheme.* ***(d)*** *Venn diagrams showing reproducibility of PPI identifications between HPG-enriched samples from both experiments.* ***(e)*** *Literature review of detected virus-host and virus-virus PPIs. Source data are provided.*

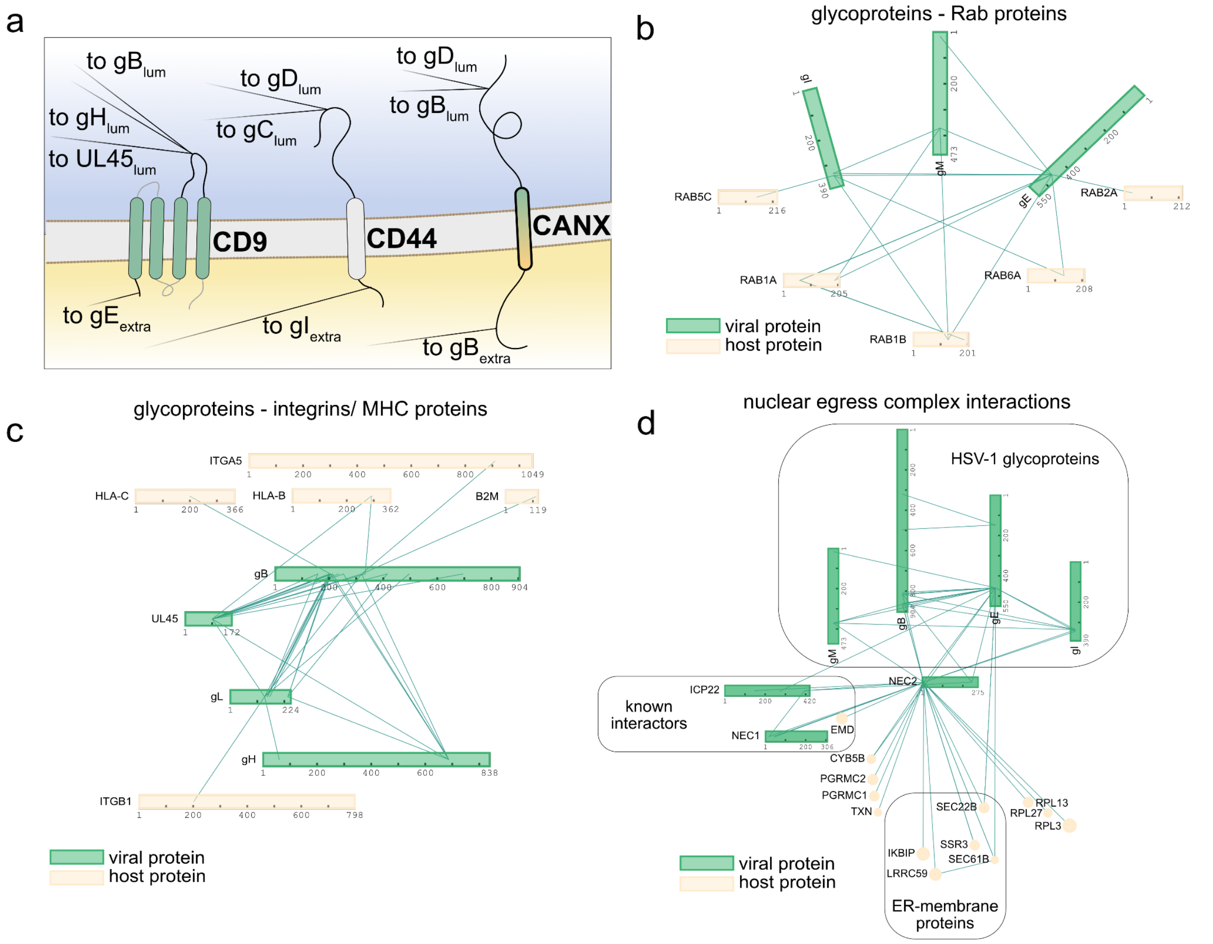

Supplementary Figure 2. Selected Subnetworks at the host membrane system ***(a)*** *Three transmembrane host proteins cross-linked to viral transmembrane proteins via their lumenal and extra-lumenal domains. Black edges indicate cross-links to viral transmembrane proteins (lum: lumenal domain, highlighted blue; extra: extra-lumenal domain, highlighted yellow). Coloring of host proteins analogous to Figure 3 e.* ***(b-d)*** *Selected subnetworks [108] of RAB proteins and their viral transmembrane protein interaction partners* ***(b)****, integrins, MHC proteins and their viral transmembrane protein interaction partners* ***(c),*** *and NEC2 with cellular and viral interaction partners* ***(d)****. The NEC2 interactors UL49, UL47 and US11 are not included in panel d for reasons of visual clarity. Source data are provided.*

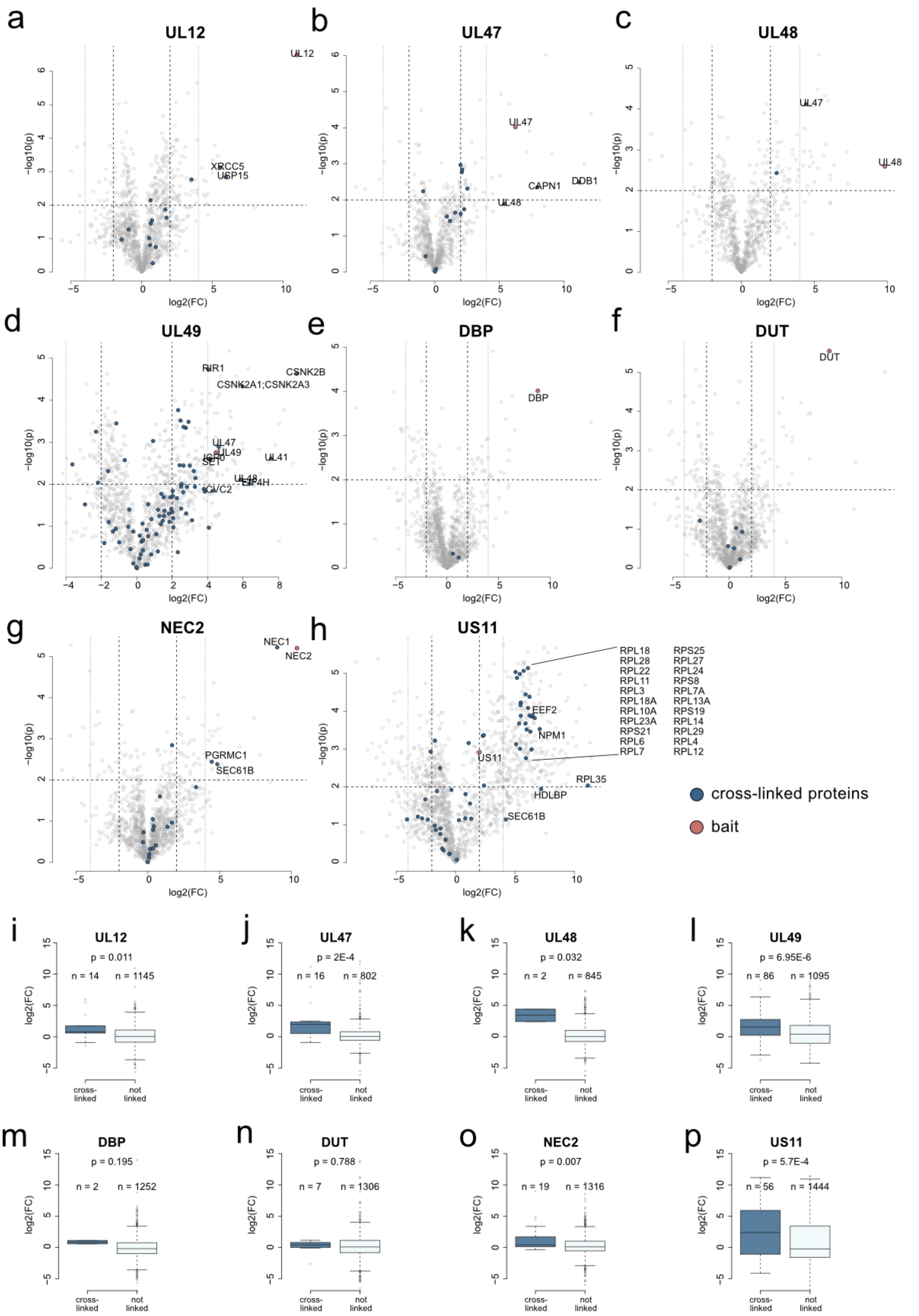

Supplementary Figure 3. Volcano plot analysis of AP-MS experiments*. *(a-h) *Each volcano plot gives the log2 fold-change as an estimate of the effect size (x-axis) and -log10 P-value (y-axis) as an estimate for the significance for protein interactions*

to a specific bait. P-values are based on two-sided t-tests without multiple hypothesis correction and n=3 biological replicates. Proteins cross-linked to the respective bait when co-enriched with a log2 fold-change > 4 are highlighted and labeled with their gene names. ***(i-p)*** *Average co-enrichment levels of cross-linked or non-cross-linked proteins to the bait for the individual AP-MS experiments. P-values are based on two-sided wilcoxon rank sum tests (center line, median; box limits, upper and lower quartiles; whiskers, 1.5× interquartile range, dots outside whiskers: outliers). Source data are provided.*

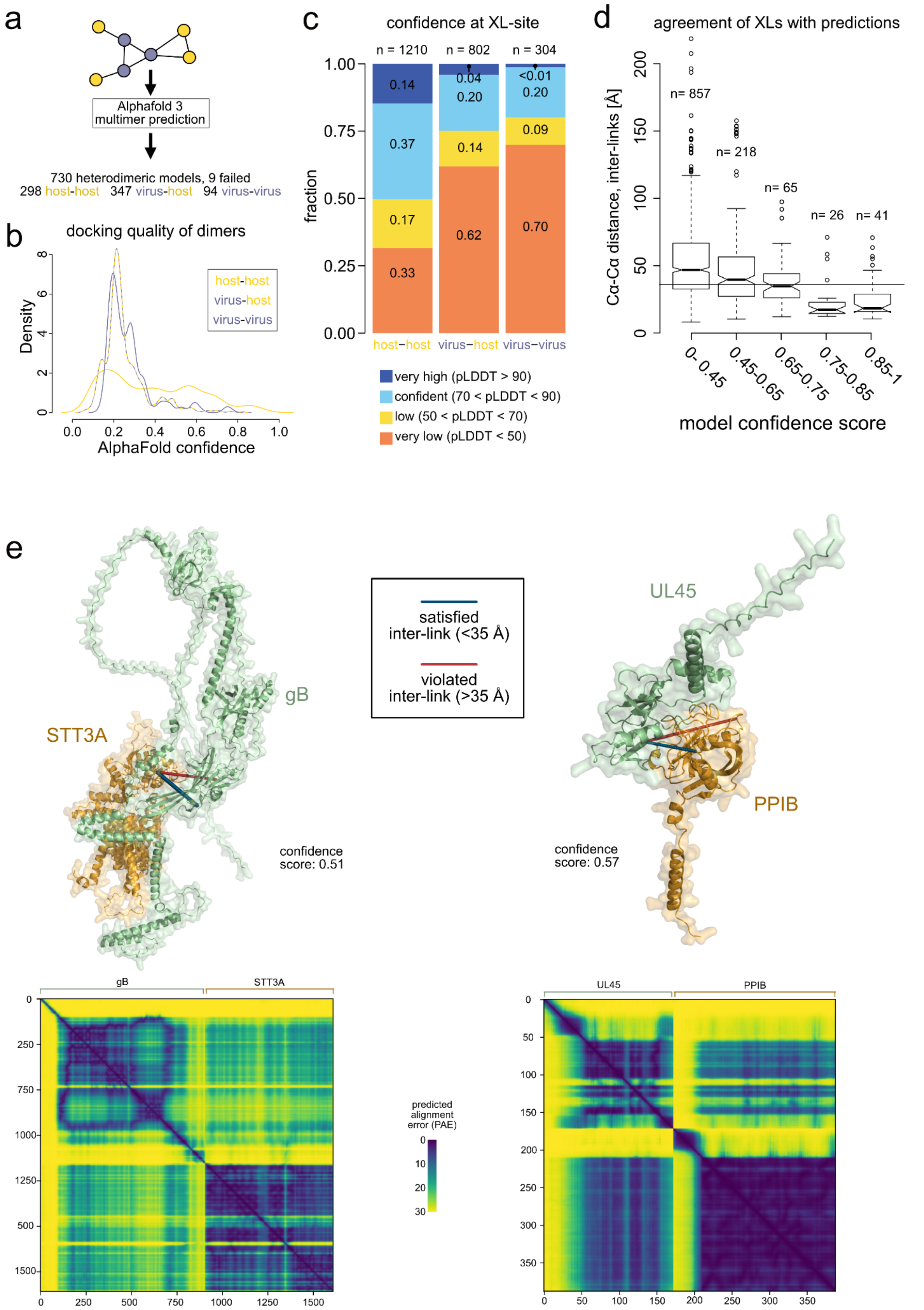

Supplementary Figure 4. Structural insight into virus-host PPIs through SHVIP and AF3 (a,b)** Predicting virus-virus, virus-host and host-host dimers of the structural interactome using AF3 **(a)** with their respective confidence score distributions **(b)**. **(c)

Per-residue prediction confidence (AF3 pLDDT score) at the specific inter-linked lysine residue for different types of inter-links. The lower pLDDT on either lysine residue of the cross-link was used for categorization. ***(d)*** *Distribution of inter-link distances (Cα-Cα distance of cross-linked lysines) for AF3 models in different confidence score ranges. Shown are only inter-links involving lysines from protein regions with pLDDT scores > 50. Boxes represent lower and upper quartiles with median marked as horizontal line. Whiskers represent 1.5 times interquartile range.* ***(e)*** *Examples of two heterodimeric models that met inclusion criteria based on confidence score and percentage of violated cross-links. See also Figure 4 for AF2 data. Source data are provided.*

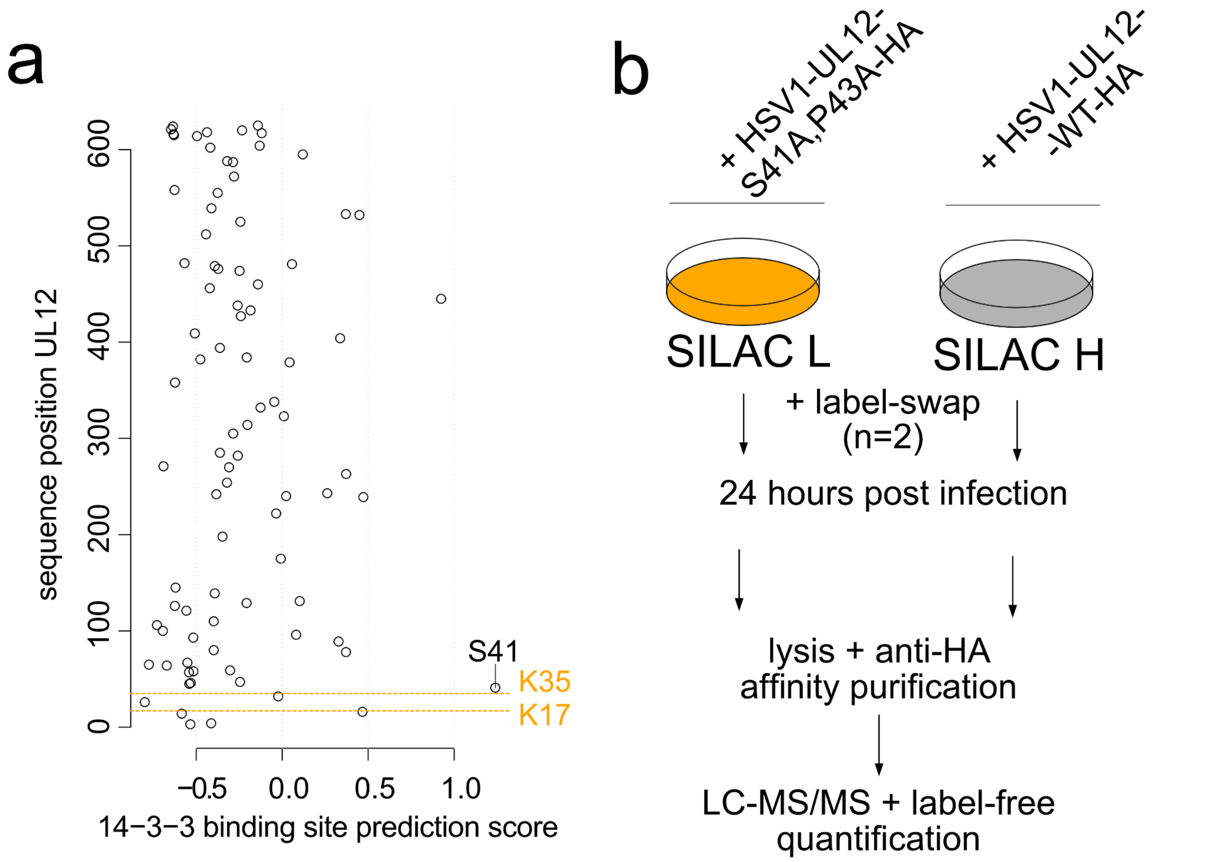

***Supplementary Figure 5. Interaction of 14-3-3 with UL12**. **(a)** Prediction of 14-3-3 binding site consensus score along the UL12 primary sequence based on 14-3-3pred [78]. The best-scoring site S41 and the lysines cross-linked to 14-3-3 are highlighted. **(b)** Experimental design for SILAC-based comparative AP-MS of widltype-UL12 to S41A,P43A-UL12. Source data are provided.*

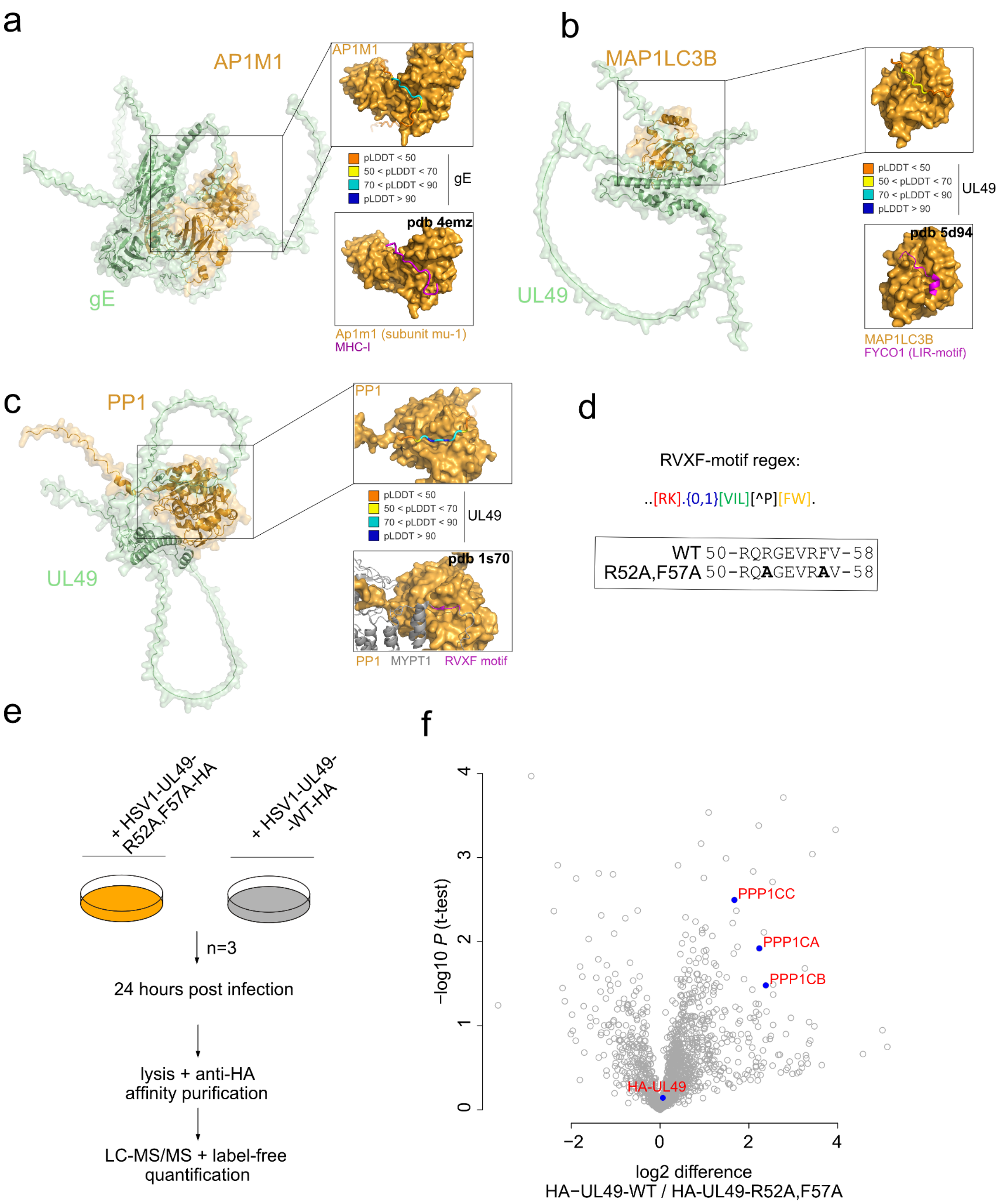

Supplementary Figure 6. Interactions mediated through IDRs. (a-c) *Examples of interactions putatively mediated through IDRs of gE-AP1M1 (a), UL49-MAP1LC3B (b) and UL49-PPP1CA (c). Insets show confidence of AF2 prediction based on pLDDT. Comparisons to experimentally solved structures are shown below the insets.* ***(d)*** *Regular expression for RVXF-type interaction motifs to PP-1. Boxed is the amino acid sequence of UL49 having a non-canonical RVXF-motif. Mutation of the non-canonical RVXF-motif in UL49 creates R52A,F57A-UL49.* ***(e)*** *Experimental design for label-free AP-MS experiments comparing HSV1-HA-UL49-WT to HSV1-HA-UL49-R52A,F57A. Experiment was performed in n=3 biological replicates.* ***(f)*** *AP-MS experiments directly comparing the interactome of wildtype UL49 to mutant UL49 in a label-free set-up based on n=3 biological replicates from infected cells. The bait (HA-UL49) and the*

three different catalytic subunits of PP-1, which can all interact with RVXF-type motifs, are labeled. P-values are based on two-sided t-test without multiple hypothesis correction. Source data are provided.
