# Supplementary material for: Structural host-virus interactome profiling of intact infected cells
Source: Nat Commun. 2025 Jul 21;16:6713. doi: 10.1038/s41467-025-61618-z (PMC12280212; doi:10.1038/s41467-025-61618-z)
Supplement: Supplementary file 2 — Description of Additional Supplementary Files [file 41467_2025_61618_MOESM2_ESM.pdf]

Description of Additional Supplementary Files

File Name: Supplementary Data 1

Description: Quantification and Identification of the proteome from HPG-enriched and input samples based on linear (non-cross-linked) peptides.

File Name: Supplementary Data 2

Description: The structural interactome of HSV-1 with the cross-link data in sheet 1 and corresponding PPI data in sheet 2.

File Name: Supplementary Data 3

Description: AP-MS data (replicate LFQ values, p-values based on two-sided t-tests without multiple hypothesis correction and average fold-changes) of 8 selected baits (sheet 1-8) from HSV1 infected cells at 24 hpi.

File Name: Supplementary Data 4

Description: Dimeric models with at least 50% satisfied inter-links taking into account only cross-links from well structured regions (AlphaFold2 sheet 1, AlphaFold3 sheet 3) and distance measurements of corresponding inter-links (AlphaFold2 sheet 2, AlphaFold3 sheet 4).

File Name: Supplementary Data 5

Description: Ordered predicted PPI interfaces within disordered regional context. Predicted interaction sites were manually curated taking into account known binding modes and structures, sequence similarity, subcellular location and prediction confidence. Sites not meeting these criteria are labeled as 'rejected'.

File Name: Supplementary Data 6

Description: Primer sequences used for BAC mutagenesis.

File Name: Supplementary Data 7

Description: Allocation of different MS-based experiments conducted in this Manuscript with corresponding Figures and search files plus raw files as uploaded into the proteomics identifications database.
